# Supplementary material for: Patient-specific therapeutic benefit of MuSK agonist antibody ARGX-119 in MuSK myasthenia gravis passive transfer models
Source: iScience. 2024 Dec 21;28(2):111684. doi: 10.1016/j.isci.2024.111684 (PMC11783450; doi:10.1016/j.isci.2024.111684)
Supplement: Document S1. Figures S1–S8 and Tables S1–S3 [file mmc1.pdf]

iScience, Volume *28*

Supplemental information

Patient-specific therapeutic benefit of MuSK agonist antibody ARGX-119 in MuSK myasthenia gravis passive transfer models

Jamie L. Lim, Stine Marie Jensen, Jaap J. Plomp, Bernhardt Vankerckhoven, Christa Kneip, Rani Coppejans, Christophe Steyaert, Kathleen Moens, Lieselot De Clercq, Martijn R. Tannemaat, Peter Ulrichts, Karen Silence, Silvère M. van der Maarel, Dana L.E. Vergoossen, Roeland Vanhauwaert, Jan J. Verschuuren, and Maartje G. Huijbers

Table S1: Patient characteristics, related to STAR Methods and Figure 1.

Patient IgG4	Plasmapheresis material composition	Sex & ethnicity	Age at MuSK MG onset	Age at time of plasmapheresis	MGFA grade at time of plasmapheresis	Treatment	MuSK reactivity (nM)
1	Pooled from two plasmaphereses	M, Caucasian	30	53 & 57	3B	Pyridostigmine, Prednisone, Mycophenolate	182
2	Pooled from two plasmaphereses	M, Caucasian	19	22	2B	Pyridostigmine, Prednisone, Azathioprine, IvIgG (unsuccesful)	607
3	One plasmapheresis session	M, Caucasian	67	69	3B	None	142
4	One plasmapheresis session	M, Caucasian	53	53	2	Prednison Azathioprine	99

M, male.

Table S2: **SPR data quantifying competition of ARGX-119 and patient IgG4 for MuSK-Fz domain binding, related to Figure 3.**

	**Mouse Fz-domain**		**Human Fz-domain**	
**Material**	**Binding of IgG4 alone (RU)**	**%Competition with ARGX-119**	**Binding of IgG4 alone (RU)**	**% competition with ARGX-119**
**Healthy donor IgG4**	0	0	0	0
**Pt 1 IgG4**	4	0	10	0
**Pt 2 IgG4**	33	0	109	83
**Pt 3 IgG4**	0	0	2	0
**Pt 4 IgG4**	0	0	10	0

Table S3: Quantification of IgG subclasses in purified IgG4 batches quantified as a percentage of the sum of the subclasses, related to Figure 3.

	**IgG1 (% of sum of subclasses)**	**IgG2 (% of sum of subclasses)**	**IgG3 (% of sum of subclasses)**	**IgG4 (% of sum of subclasses)**
**Pt 1**	3	3	0.1	94
**Pt 2**	3	3	0.1	94
**Pt 3**	7	2	0.1	91
**Pt 4**	4	6	0.1	90

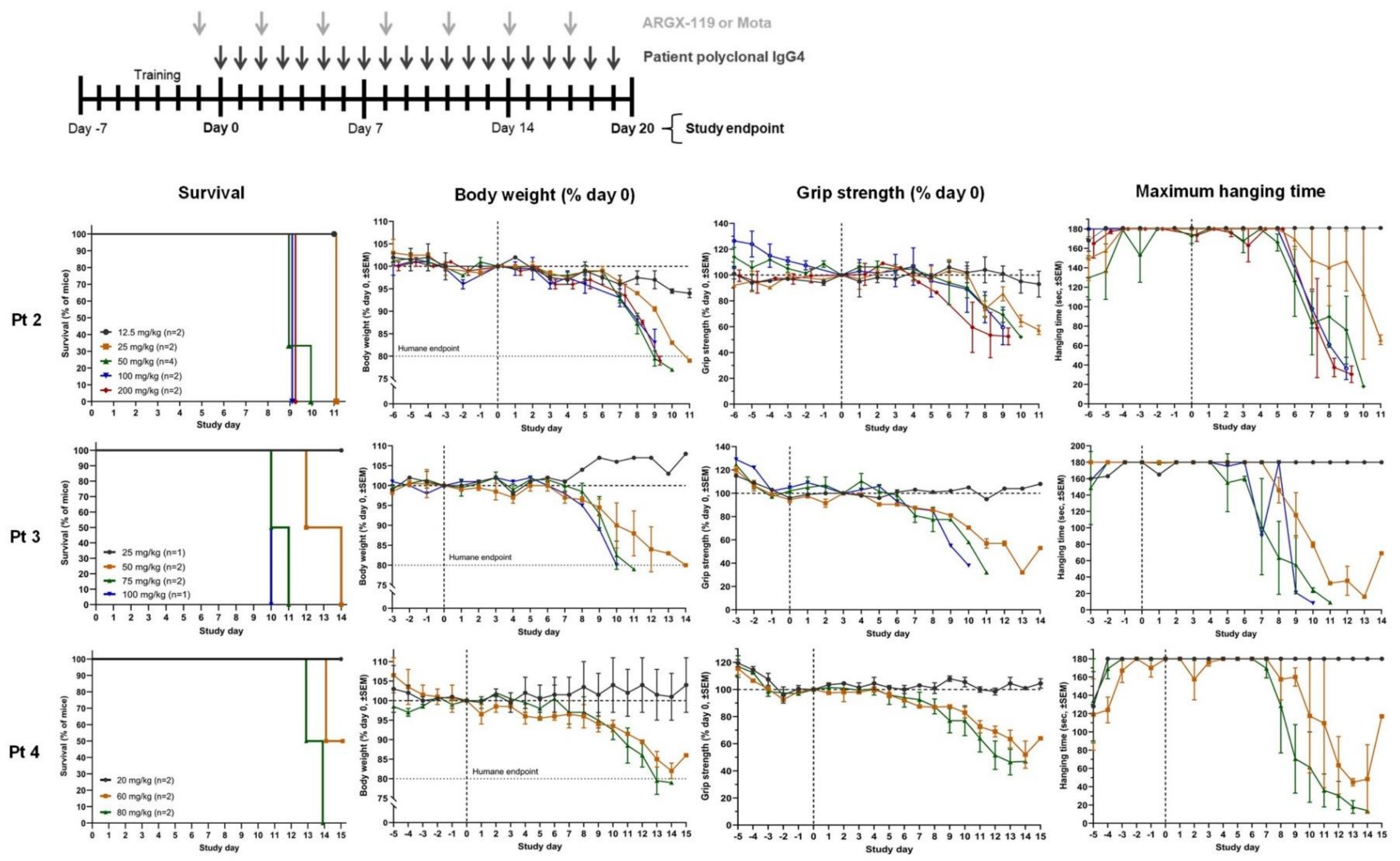

Figure S1: MG phenotype dose findings of purified patient IgG4 in mice. *From left to right for patients 2-4*: percentage mouse survival over time, with reductions in survival representing sacrifice of mice due to humane endpoint reached; percentage body weight normalized to day 0; percentage grip strength normalized to day 0; and

hanging time (maximum 180 seconds, best of 3 attempts) in mice treated with different doses of each purified patient IgG4. The doses in orange were identified as the minimal dose for a maximal MG phenotype per patient material. Data points of percentage grip strength and hanging time represent grouped values of the mean ±SEM of mice.

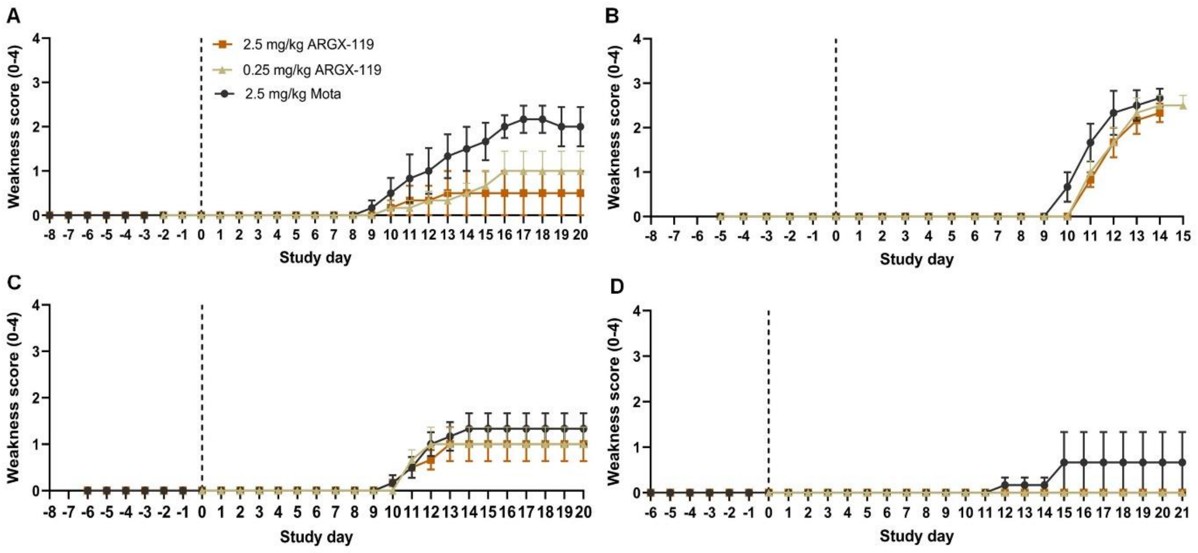

Figure S2: ARGX-119 improves clinical scores in the MuSK MG mouse model induced with polyclonal patient IgG4 in a patient-specific manner. Stacy clinical scores (0 = no weakness, 1 = weakness upon activity, 2 = weakness at rest, 3 = severe weakness with breathing difficulty, 4 = death) (Stacy et al., 2002) in mice treated with 2.5 or 0.25 mg/kg ARGX-119 or 2.5 mg/kg isotype control Mota and with (A) Patient 1, (B) Patient 2, (C) Patient 3 or (D) Patient 4 IgG4. Data points represent grouped values of the mean ±SEM of mice. In case of missing data due to mouse sacrifice, the last value of each mouse was carried forward until the end of the experiment.

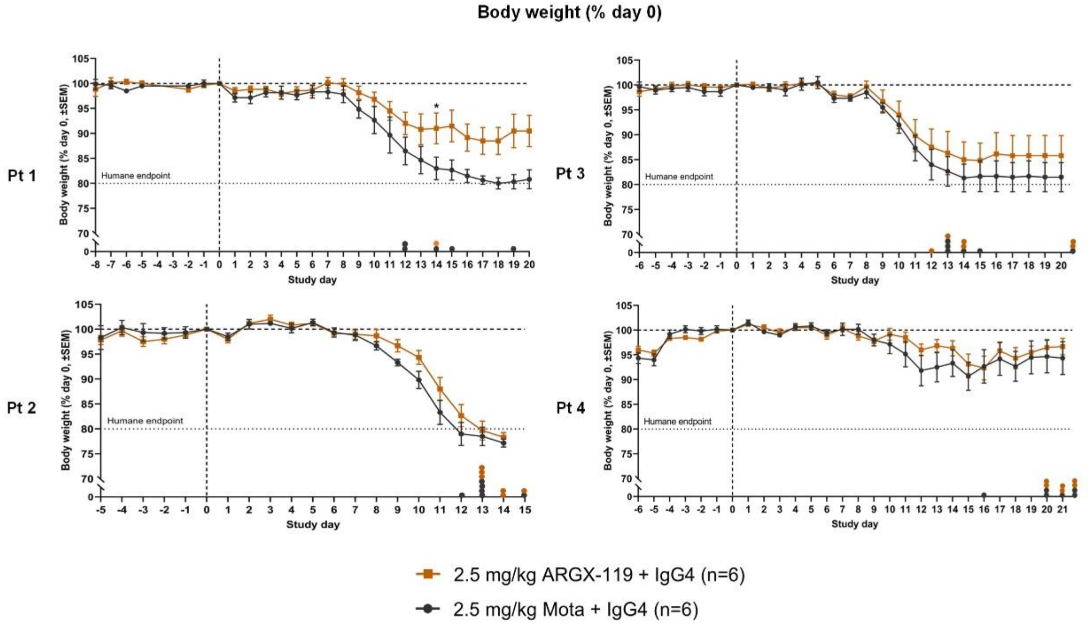

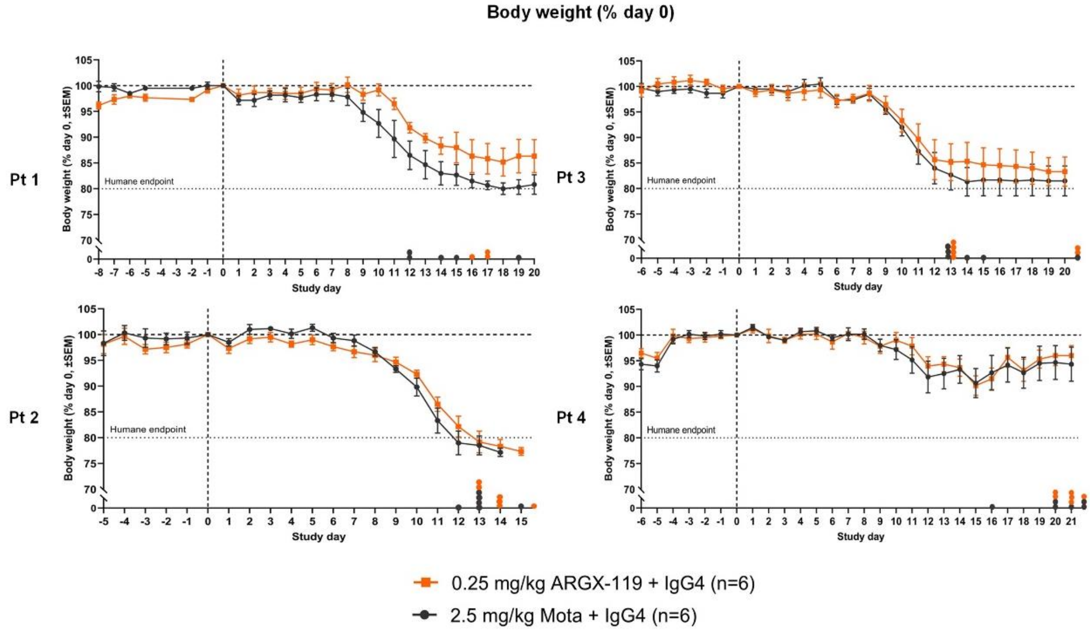

Figure S3: ARGX-119 improves body weight in the MuSK MG mouse model induced with polyclonal patient IgG4 in a patient-specific manner. Percentage body weight normalized to day 0 in mice treated with 2.5 or 0.25 mg/kg ARGX-119 or 2.5 mg/kg isotype control Mota. Data points of percentage body weight represent grouped values of the mean ±SEM of mice. Data points on the x-axis represent mice that were sacrificed as they reached humane endpoint or end of study. Dotted horizontal line represents the humane endpoint (80% body weight of day 0). Statistical analyses were only performed on average data points when the groups contained more than three mice. *P<0.05 for ARGX-119 vs Mota treated mice.

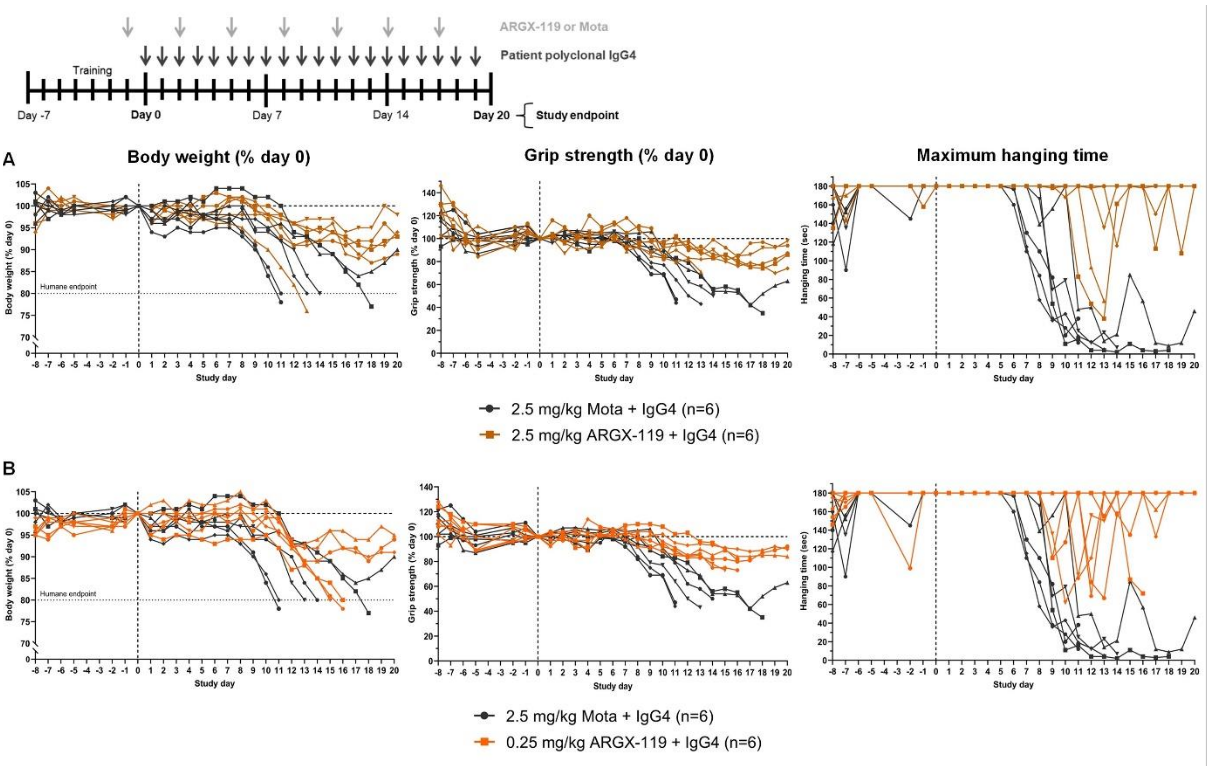

Figure S4: ARGX-119 rescues the MuSK MG phenotype in mice induced with polyclonal IgG4 from patient 1. A) *From left to right*: percentage body weight normalized to day 0 of mice (threshold of humane endpoint depicted at 80% body weight); percentage grip strength normalized to day 0; hanging time (maximum 180 seconds, best of 3 attempts) of mice treated with A) 2.5 mg/kg ARGX-119 or B) 0.25 mg/kg ARGX-119, versus isotype control 2.5 mg/kg Mota (n=6/group). Data points represent individual mouse values.

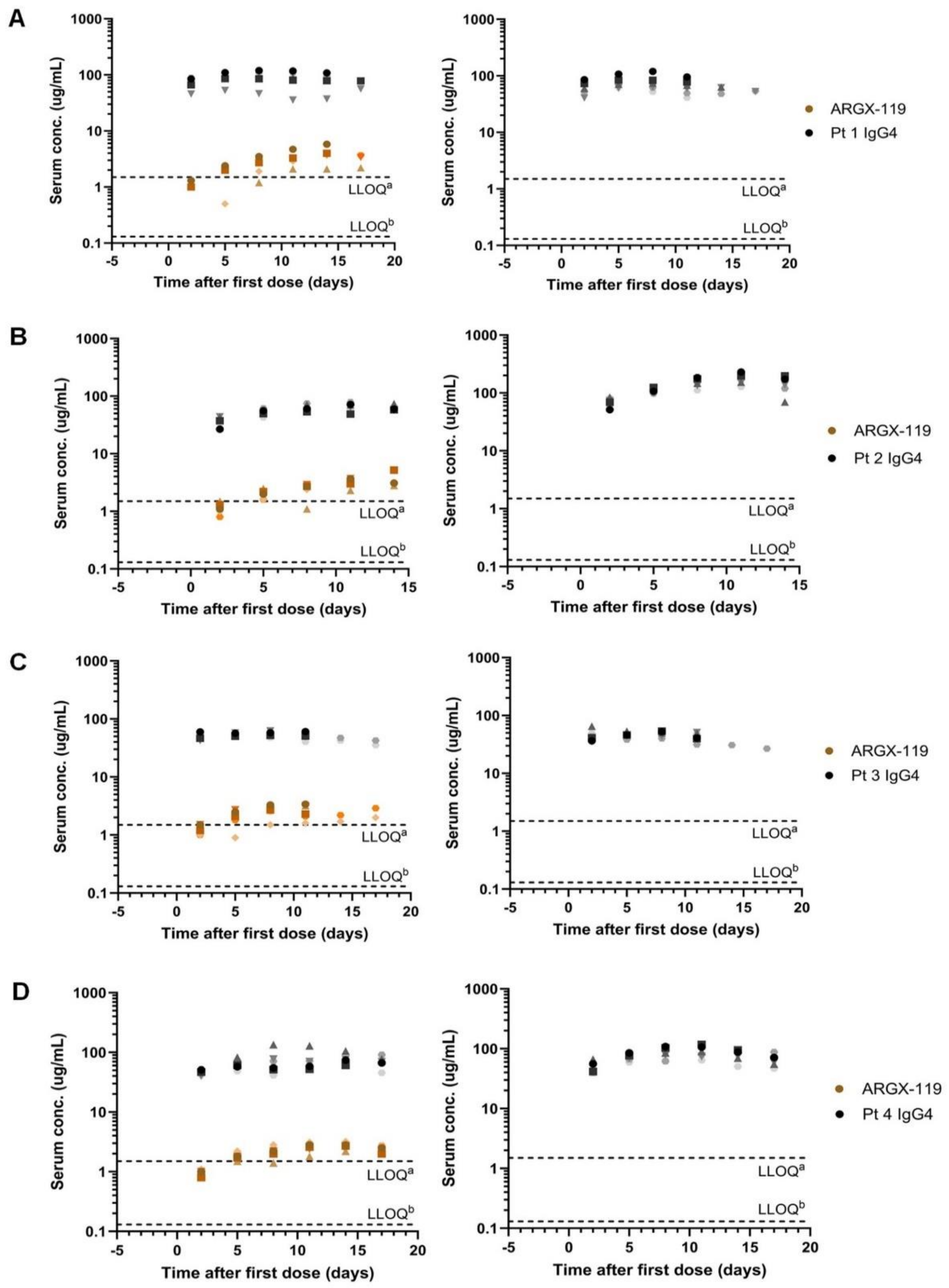

Figure S5: Exposure to ARGX-119 and polyclonal patient IgG4 was overall consistent between mice within the different experiments. Serum concentrations (ug/mL) in individual mice over time until humane endpoint or end of study before and after administration of 0.25 mg/kg ARGX-119 or 2.5 mg/kg isotype control Mota (from left to right) and A) 40 mg/kg/day patient 1 IgG4, B) 25 mg/kg/day patient 2 IgG4, C) 50 mg/kg/day patient 3 IgG4 or D) 60 mg/kg/day patient 4 IgG4. Brown-to-orange colored data points represent ARGX-119 exposure in individual mice (each mouse represented by one color) and black-to-grey colored data points represent patient IgG4 exposure in the same mice (each mouse represented by one color). LLOQ^{a}, lower limit of quantification of patient IgG4 PK; LLOQ^{b} of ARGX-119 PK.

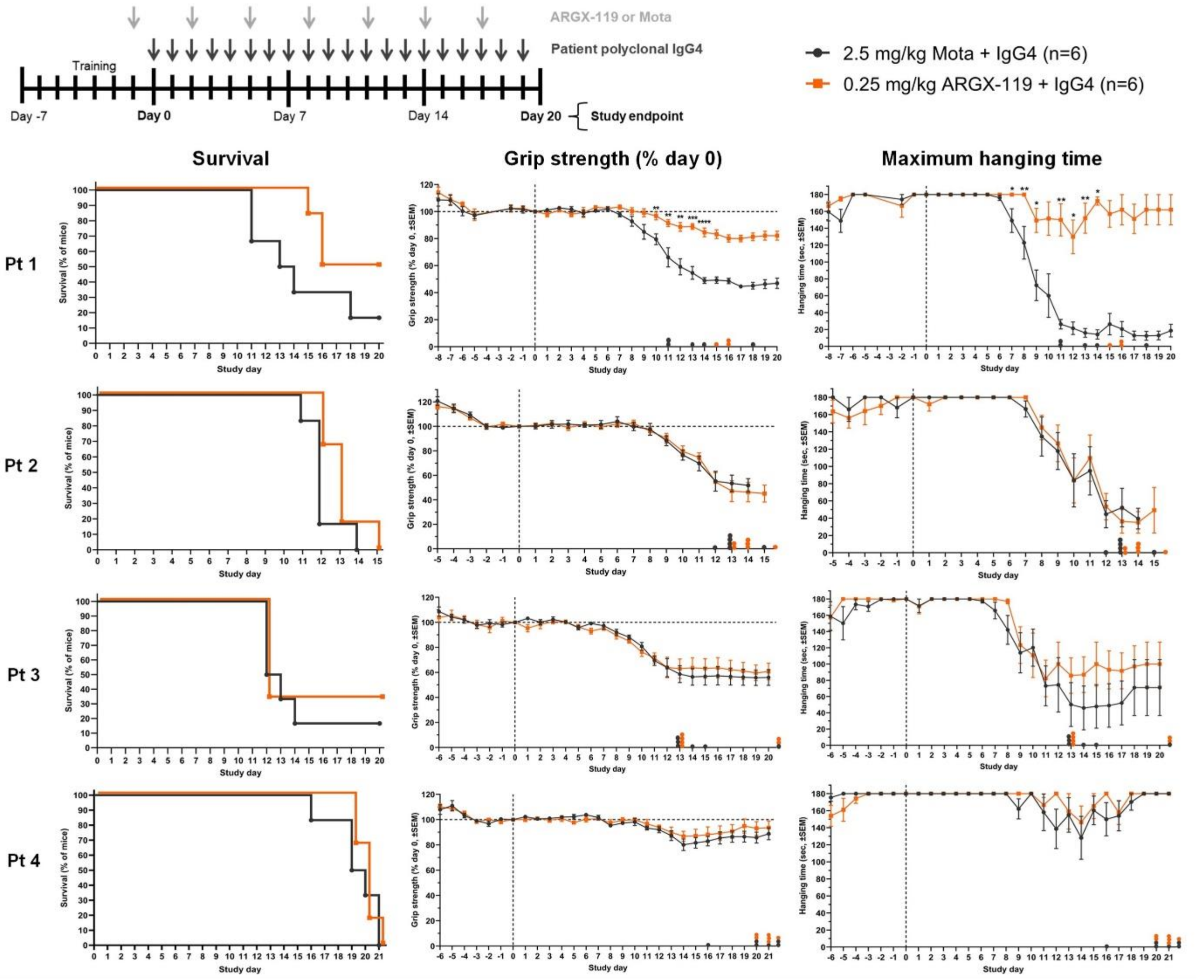

Figure S6: ARGX-119 rescues the MuSK MG phenotype induced in mice with polyclonal patient IgG4 in a patient-specific manner. *From left to right for patients 1-4*: percentage mouse survival over time, with reductions in survival representing sacrifice of mice due to humane endpoint reached; percentage grip strength normalized to day 0; and hanging time (maximum 180 seconds, best of 3 attempts) in mice treated with 0.25 mg/kg ARGX-119 or isotype control Mota. Data points of percentage grip strength and hanging time represent grouped values of the mean ±SEM of mice. Data points on the x-axis represent mice that were sacrificed as they reached humane endpoint or end of study. Statistical analyses were only performed on average data points when the groups contained more than three mice. *P<0.05, **P<0.01, ***P<0.001, ****P<0.0001 for ARGX-119 vs Mota treated mice.

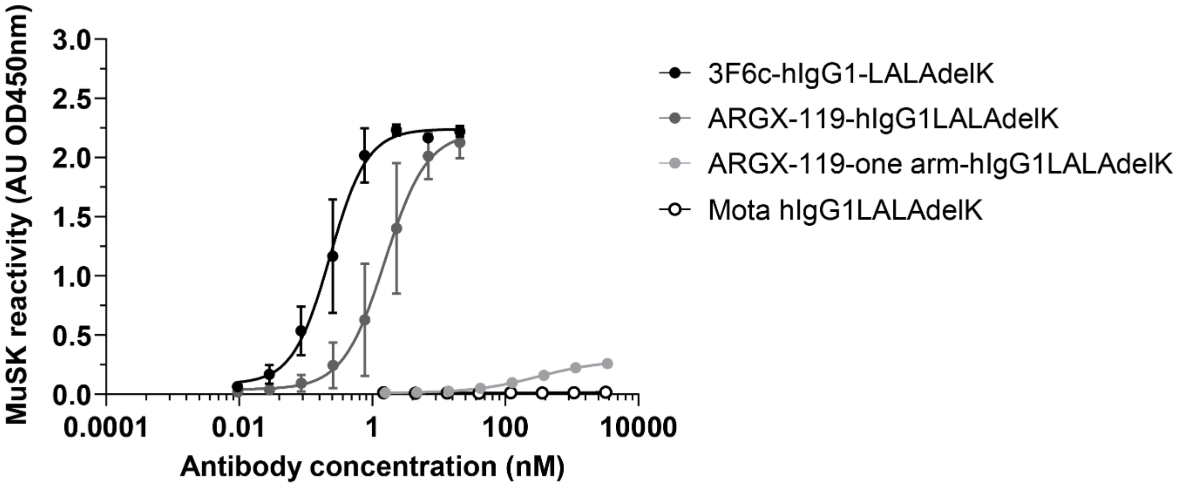

Figure S7: Confirmation of the MuSK bio-bridging assay design. Bivalent MuSK antibodies 3F6c-hIgG1-LALAdelK and ARGX-119 hIgG1LALAdelK serve as positive controls in this bio-bridging assay, whereas a one-armed ARGX-119 antibody and the isotype control antibody Mota show no to little binding in this assay.

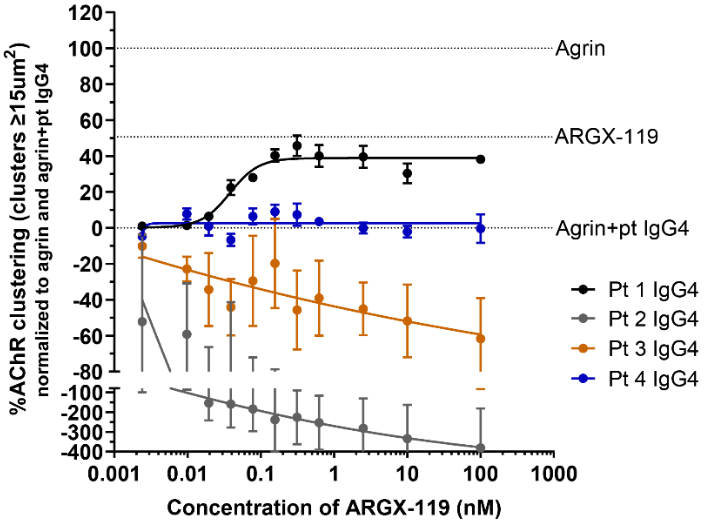

Figure S8: Dose-response curve of ARGX-119 in an *in vitro* MuSK MG model. Dose-response curve of ARGX-119 on AChR clustering (clusters ≥15um^2) in C2C12 myotubes treated with pt IgG4s. The dashed lines represent conditions where agrin or ARGX-119 were added to the cultures alone. The data was related to the inhibitory capacity of each of the patient IgG4s individually. Data represent mean ± SEM.
